# Human Papillomavirus 16 E6 and E7 Oncoproteins Alter the Abundance of Proteins Associated with DNA Damage Response, Immune Signaling and Epidermal Differentiation

**DOI:** 10.3390/v14081764

**Published:** 2022-08-12

**Authors:** Kerry Dust, Michael Carpenter, Julie Chih-yu Chen, Chris Grant, Stuart McCorrister, Garret R. Westmacott, Alberto Severini

**Affiliations:** 1 Department of Medical Microbiology, Faculty of Health Sciences, University of Manitoba, Winnipeg, MB R3E 0W2, Canada; 2 Cadham Provincial Laboratory, Manitoba Health, Winnipeg, MB R3C 3J7, Canada; 3 National Microbiology Laboratory, Public Health Agency of Canada, Winnipeg, MB R3E 3M4, Canada

**Keywords:** human papillomavirus, E6 protein, E7 protein, proteomics, keratinocytes, transformation

## Abstract

The high-risk human papillomaviruses are oncogenic viruses associated with almost all cases of cervical carcinomas, and increasing numbers of anal, and oral cancers. Two oncogenic HPV proteins, E6 and E7, are capable of immortalizing keratinocytes and are required for HPV associated cell transformation. Currently, the influence of these oncoproteins on the global regulation of the host proteome is not well defined. Liquid chromatography coupled with quantitative tandem mass spectrometry using isobaric-tagged peptides was used to investigate the effects of the HPV16 oncoproteins E6 and E7 on protein levels in human neonatal keratinocytes (HEKn). Pathway and gene ontology enrichment analyses revealed that the cells expressing the HPV oncoproteins have elevated levels of proteins related to interferon response, inflammation and DNA damage response, while the proteins related to cell organization and epithelial development are downregulated. This study identifies dysregulated pathways and potential biomarkers associated with HPV oncoproteins in primary keratinocytes which may have therapeutic implications. Most notably, DNA damage response pathways, DNA replication, and interferon signaling pathways were affected in cells transduced with HPV16 E6 and E7 lentiviruses. Moreover, proteins associated with cell organization and differentiation were significantly downregulated in keratinocytes expressing HPV16 E6 + E7. High-risk HPV E6 and E7 oncoproteins are necessary for the HPV-associated transformation of keratinocytes. However their influence on the global dysregulation of keratinocyte proteome is not well documented. Here shotgun proteomics using TMT-labeling detected over 2500 significantly dysregulated proteins associated with E6 and E7 expression. Networks of proteins related to interferon response, inflammation and DNA damage repair pathways were altered.

## 1. Introduction

Human papillomaviruses (HPV) are small, non-enveloped DNA viruses, which infect epithelial keratinocytes. These viruses are transmitted by physical contact, resulting in warts, hyperplastic lesions and, in some cases, cancer [1]. Of the hundreds of types of HPV, there is a small group classified as high-risk for their association with oropharyngeal and anogenital cancer by the International Committee on Taxonomy of Viruses [2].

Although most infections with HPV are cleared by the host within two years, infections which persist pose a high risk for carcinogenesis [3]. The long-term expression of high-risk HPV E6 and E7 oncoproteins promote immortalization and transformation of keratinocytes but the exact mechanism by which this occurs is not completely known [4,5]. The ability of high-risk HPV oncoproteins E6 and E7 to bind and degrade the host tumor suppressor proteins retinoblastoma (pRb) and p53 to promote viral replication and prevent host apoptosis is well-established [6,7,8,9]. Additionally, the E6 and E7 oncoproteins are known to degrade, mislocalize or activate many other host proteins [10,11,12,13]. A thorough characterization of the global dysregulation of the host proteome and signaling pathways affected by HPV16 E6 and E7 would clarify the roles of the E6 and E7 oncoproteins in HPV biology and determine how these viruses evade immune detection. It would additionally illuminate the mechanisms that foster persistence and lead to transformation.

Previous investigations into the effects of HPV infection revealed altered protein expression signatures compared to uninfected or non-cancerous tissues [14,15,16,17,18]. Unfortunately, most of these studies used cancerous cells or tissues and using such a heterogeneous population of cells to study proteomic changes dampens the power of those investigations to discern the effects of viral proteins on the host proteome. Additionally, any differential protein levels observed in HPV infected tissues cannot be specifically attributed to the E6 and E7 proteins. There have been previous studies investigating the effects of HPV16 E6 or HPV16 E6 and E7 on the host proteome using two-dimensional difference gel electrophoresis followed by mass spectrometry. However the limited sensitivity of gel based proteomic methods may explain the scarcity of deregulated proteins associated with those studies [19] Current shotgun proteomics analysis by liquid chromatography-tandem mass spectrometry (LC-MS/MS) coupled with pathway analysis promise enhanced sensitivity for the exploration of the global dysregulation of the host proteome by full-length HPV16 E6 and E7 proteins [20].

To catalogue the differential abundance of critical proteins altered in primary keratinocytes expressing full-length HPV16 E6 and E7 proteins, we employed gel free, liquid chromatography coupled with tandem mass spectrometry for a shotgun proteomics analysis. The use of isobaric tags is a well-established method for measuring differential protein levels between samples [20].

Here we provide a glimpse into the global proteomic alterations resulting from the presence of HPV oncoproteins. Canonical signaling pathways related to DNA damage response, DNA replication and interferon signaling pathways were affected in cells transduced with lentiviruses encoding HPV16 E6 and E7 genes. In addition, proteins associated with cell organization and differentiation were significantly downregulated in keratinocytes expressing HPV16 E6 and E7.

## 2. Methods

### 2.1. Cell Culture

Primary human neonatal keratinocytes (HEKn) (Life Technologies, Grand Island, NE, USA, cat# C0015C) were grown in Epilife media (Life Technologies, Grand Island, NE, USA, cat# MEPI500CA) supplemented with 1% human keratinocyte growth serum (HKGS) (Life Technologies, Grand Island, NE, USA, cat# S0015). Cultures were maintained at 37 °C in a humidified incubator at 5% CO_2_.

### 2.2. Lentivirus Production

A splice mutant of the HPV16 E6 gene (Genbank Accession# NC_001526) was constructed with a point mutation (nt 227 G→A) that abolishes the 5′ splice site to promote the expression of full- length E6 proteins. Truncated E6 proteins resulting from the splice site do not have the same biological capacity for transformation as the full length E6 proteins [21].

This splice-mutant HPV16 E6 gene was inserted into pCDH-CMV-MCS-EF1-Neo (Systems Biosciences, Palo Alto, CA, USA, cat# CD514B-1) lentivector, and an HPV16 E7 gene was inserted into pCDH-CMV-MCS-EF1-Hygro (Systems Biosciences, Palo Alto, CA, USA, cat# CD515B-1) lentivector.

293TN cells (Systems Biosciences, Palo Alto, CA, USA, cat#LV900A-1) were transfected with 1 mL DMEM, lentivector and packaging plasmids: pPACKH1-GAG, pPACKH1-REV, and pVSV-G along with 30 µL of Roche Xtreme gene HP (Roche, Mississauga, ON, Canada cat# 06366236001). The transfected 293TN cells were maintained in DMEM (Life Technologies, Grand Island, NE, USA, cat#11966-025) supplemented with 4 mM L-glutamine, 4 g/L glucose, 100 units/mL penicillin-G, 100 µg/mL streptomycin (90%) and 10% fetal bovine serum (FBS) for 48 h at 37 °C in a humidified incubator at 5% CO_2_.

Lentiviruses were harvested from the tissue culture supernatant, mixed with PEG-it™ viral precipitation solution (System Biosciences, Palo Alto, CA, USA, cat# LV810A-1) and stored at 4 °C overnight to concentrate the virus. The next day the lentiviruses were pelleted by centrifugation at 1500× *g* for 40 min at 4 °C. The pellet was resuspended in 1/10 the volume of DMEM supplemented with 2% FBS.

### 2.3. Lentiviral Titration

To determine the lentiviral titer, the number of functional lentiviral particles was measured by qPCR of the lentivector Woodchuck Hepatitis Virus Posttranscriptional Regulatory Element following infection of HEKn cells [22]. GAPDH was used for normalization. DNA was extracted from infected cells 48 h post-infection using the Qiagen QIAamp DNA mini kit (Qiagen, Germantown, TN, USA, cat# 51304) as per the manufacturer’s instructions. In each 20 µL reaction, 0.4 µM of each primer (WPRE Forward: CGCTGCTTTAATGCCTTTGTAT; WPRE Reverse: GGGCCACAACTCCTCATAAA) was added to 10 µL of SYBR green master mix (Biotool.com cat# B21203). A Roche 480 Lightcycler (Roche Life Sciences Inc., Indianapolis, IN, USA) was used for DNA amplification. Cycling conditions were as follows: one cycle of 50 °C for 2 min, one cycle of 95 °C for 2 min, 40 cycles of 95 °C for 3 s and 60 °C for 30 s. Similarly, for GAPDH amplification 0.4 µM of primers and 10 µL of SYBR green were used in each 20 µL reaction PCR: (GAPDH Forward: ACATCGCTCAGACACCATG; GAPDH reverse: TGTAGTTGAGGTCAATGAAGGG). The titer of each sample was then determined by calculating the amount of WPRE element relative to that of GAPDH DNA against a standard curve that was generated from serially diluted WPRE DNA quantified standards.

### 2.4. Transduction

HEKn cells at approximately 25 population doublings were seeded into 24 well culture plates (Corning, Corning, NY, USA, cat# 3524) at 25,000 cells per well. HEKn cells were transduced with both HPV16 E6 and E7 lentiviruses at an MOI of one. For controls, HEKn cells were transduced with lentiviruses created with unmodified lentivectors (LV-HEKn). Four biological replicates were performed for the HPV16 E6 and E7, and the LV-HEKn transductions. To control for transduction, and antibiotic selection, HEKn cells were transduced with lentiviruses carrying a Green Fluorescent Protein (GFP) insert. The transduced cells were incubated at 37 °C in 5% CO_2_. Antibiotic selection was started at the first media change, 48 h post-transduction. Initially, 15 μg/mL Gibco Geneticin G418 Sulfate (Thermo Fisher Scientific, Waltham, MA, USA, cat # 10131035) and 15 μg/mL Hygromycin B (Thermo Fisher Scientific, Waltham, MA, USA, cat # 10687010) were used for five days. Then, 5 μg/mL of each were used for ten days to ensure the elimination of cells not containing a lentivirus insertion. Four biological replicates were performed in a similar manner.

The presence of HPV16 E6 and E7 mRNA was verified by RT-PCR. First E6 and E7 mRNA was extracted from the transduced cells using Qiagen’s RNeasy kit (Qiagen cat# 74104) as per the manufacturer’s instructions. Each RT-PCR reaction consisted of 5 µL of TaqMan^®^ Fast Virus 1-Step Master Mix (TFV) (Thermo Fisher cat# 4444432), 6 µL of water and 1 nM each HPV16_E6 primers forward: CCATGCACCAAAAGAGAACTG and HPV16_E6 reverse: TCAGGACACAGTGGCTTTTGA, 5 µL of 10 ng/µL RNA. Amplification was achieved with the following conditions: 50 °C for 5 min, 95 °C for 20 s followed by 40 cycles of 95 °C for 3 s and 55 °C for 30 s. Similarly, E7 mRNA was identified using HPV16_E7 primers forward: ACATGGA TCCGCCACCATGCAT GGAGATACACC and HPV16_E7 reverse ATGTGCGGCCGCTTATTATGGTTTCTGAG. The bands were visualized using a Qiaxcel High-Resolution DNA kit (Qiagen cat# 929002) as per the manufacturer’s instructions.

### 2.5. Protein Harvest and Quantification

After selection with antibiotics, transduced cells were grown to confluence in T75 flasks. After 45 population doublings cells were harvested. Epilife media was removed and the cells were washed twice with cold PBS. Next, the cells were manually scraped from the flasks and collected in 2% Sodium dodecyl sulfate (SDS) in Phosphate-buffered saline (PBS). Cells in 2% SDS were heated for 5 min at 99 °C and then homogenized using Qiashredder column as per the manufacturer’s instructions (Qiagen, Germantown, TN, USA, cat#79656). Proteins were quantified using a Pierce BCA kit as per the manufacturer’s instructions (Pierce, Rockford, IL, USA, cat #23227).

### 2.6. Peptide Digestion and Tandem Mass Tag (TMT) Labeling

To prepare the peptides for mass spectrometry, 100 μg of proteins were combined 1:10 with 1 M Dithiothreitol (DTT) (Sigma Aldrich, St. Louis, MO, USA, cat# DO632) and heated for 5 min at 99 °C to reduce disulfide bonds. Once cooled to room temperature the sample was incubated with an equal volume of urea exchange buffer (UEB) at room temperature for 10 min. UEB is 8 M Urea (GE Healthcare, Chicago, IL, USA, cat# 17-1319-01) in 50 mM HEPES buffer (Thermo Fisher Scientific, Waltham, MA, USA, cat# 15630106). Nanosep 10 K Omega filter cartridges (PAL Life Sciences Port, Washington, DA, USA, cat# OD010C34) were prepared by adding 200 µLwater to the filter and centrifuging 10,000× *g* for 3 min followed by 200 µLUEB to the filter cartridge and centrifuge 10,000× *g* for 3 min. The samples were applied to the filter cartridge and centrifuged 10,000× *g* for 3 min × *g* for 10–15 min. The filter cartridge was washed 3 times by adding 250 µLUEB and centrifuging at 10,000× *g* for 10–15 min for each wash. After the third wash 100 µL of 50 mM Iodoacetamide (IAA) (Sigma Aldrich, St. Louis, MO, USA, cat# I6125) solution was added to the cartridge, the samples shaken at 600 rpm for 1 min, then placed in the dark for 20 min. the samples were then centrifuged at 10,000× *g* for 10–15 min. The filter cartridges were washed three times by adding 100 µLUEB and centrifuging at 10,000× *g* for 10–15 min. for each wash. The samples were further washed by adding 150 µL Ammonium Bicarbonate (AB) (Fisher Scientific, Ottawa, Canada cat# A643) and centrifuging at 10,000× *g* for 10–15 min two times. To remove any nucleic acid from the samples 50 µL of benzonase solution (2 mM MgCl2, 100 units benzonase (EMD Millipore/Novagen, St. Louis, MO, USA, cat# 70746-3) in 50 mM HEPES (pH8.3) was added to the filters. They were shaken at 600 rpm for 2 min and incubated for 30 min at room temperature. 100 µL of 50 mM HEPES pH8.3 was then added to the filters and then centrifuged at 10,000× *g* for 10–15 min. The wash was repeated 2 more times. To digest the proteins in the samples, 50 µL of trypsin solution (1.5 µg of trypsin protease MS Grade) (Pierce, Rockford, IL, USA, cat# 90058) in 50 mM HEPES pH8.3) was added to each filter to the cartridge. The samples were mixed at 600 rpm for 1 min, then incubated overnight at 37 in a humidified chamber. The next day 50 µL of 50 mM HEPES pH 8.3 was added to the filter and it was shaken at 600 rpm for 2 min at room temperature. Next, the filter was inverted into a low bind 1.5 mL Eppendorf tube (Eppendorf, Framingham, MA, USA, cat# 02-681-320) and centrifuged 10,000× *g* for 3 min to collect the peptides. This was repeated 2 more times. The wash solution containing the tryptic peptides was then concentrated by drying to 1–5 µL using a vacuum centrifuge (speed-vac).Peptides were labeled with TMT six-plex Isobaric Label Reagent Set (Thermo Fisher Scientific, Waltham, MA, USA, cat# 90061) as per the manufacturer’s protocol, and labelled samples for each TMT experiment were mixed one-to-one.

### 2.7. Liquid Chromatography and Mass Spectrometry (LC-MS/MS)

Samples were fractionated into 12 fractions by high-pH C18 reversed-phased liquid chromatography using an Agilent 1200 liquid chromatography system (Agilent Technologies, Santa Clara, CA, USA). Each fraction was concentrated to near-dryness using vacuum centrifugation, resuspended in 80 µL of buffer A (2% acetonitrile, 0.1% formic acid) and loaded (2 µL) onto a on a low-pH C18 reversed-phase Easy-Spray column (Thermo Fisher Scientific, Waltham, MA, USA; 50 cm × 100 µm) using an Easy nLC 1000 coupled to a Q-Exactive Plus mass spectrometer (Thermo Fisher Scientific, Waltham, MA, USA). Peptides were eluted off the column using linear gradients from 5% to 32% buffer B (98% acetonitrile, 0.1% formic acid) over 120 min at a constant flow of 200 nL/min. Total LC-MS/MS run-time per fraction was 160 min.

Two TMT runs were performed on a total of 4 biological replicates for each cell line. The first LC-MS/MS run included biological replicates 1 and 2 of: HPV16 E6 + E7, and HEKn control cells (HEKn transduced with empty lentivectors) and the second LC-MS/MS run included biological replicates 3 and 4. Isobaric tags were shuffled between replicate runs to avoid bias.

### 2.8. Data Analysis

A data-dependent acquisition method was used; dynamically choosing the top 15 abundant precursor ions for fragmentation by HCD. The peptide sequence data was searched against a database containing the SwissProt database (2015_04) restricted to Human (20,216 entries) and HPV (4 entries) using Mascot v2.5 (Matrix Science). Mascot search results were imported into Scaffold Q+ v4.4 (Proteome Software) and filtered using 0.1% FDR for peptides, 1.0% FDR for proteins, and at least two peptides per protein. Median normalization, inter-run and intra-run, peptide spectrum normalization was generated using Scaffold Q+ v4.4.

Further data processing included removing proteins that were not detected in any of the TMT experiments as well as any proteins that were detected in one TMT run but not the other. Principal component analysis was used for data exploration of log_2_ protein intensity while a heatmap was generated to evaluate hierarchical clustering of samples [23]. Statistical analyses for differential protein abundance were conducted using the LIMMA (Linear Models for Microarray Data) R package on log_2_ normalized protein intensity generated by Scaffold [24]. The *p*-values reported in the manuscript are adjusted by Benjamini-Hochberg (BH) method to correct for multiple hypothesis testing [25]. The threshold for significance was set at an adjusted *p*-value of ≤0.02.

To explore the protein interaction networks, biological functions and pathway analyses enriched in our dataset, we employed Ingenuity Pathway analyses (IPA) (QIAGEN Inc., https://www.qiagenbioinformatics.com/products/ingenuitypathway-analysis, accessed on 10 July 2022). STRING v10.5 (Search Tool for the Retrieval of Interacting Genes/Proteins) (accessed 4 August 2022) was used to visualize patterns among the protein networks and biological functions [26].

The IPA analyses were restricted to the 381 proteins significantly upregulated (*p*-value of ≤0.02) by at least 1.5-fold (0.6 log_2_) change and the 270 proteins significantly downregulated (*p*-value of ≤0.02) by at least 1.5-fold (0.6 log_2_) change in keratinocytes transduced with HPV16 E6 and E7 lentiviruses compared to the LV-HEKn control keratinocytes.

### 2.9. Immunoblotting

For Western blot analyses, 20 μg of total cellular proteins were added to each well of a 4–20% Mini-PROTEAN^®^ TGX™ Precast Gel (Biorad, Mississauga, Canada cat# 4561093EDU) and electrophoresed at 95 volts for 5 min followed by 200 volts for 30 min. Proteins were transferred to a nitrocellulose membrane using the iBlot2 transfer stacks (Invitrogen, Waltham, MA, USA, cat#IB23001) and Invitrogen’s iBlot system. LI-COR TBS Odyssey buffer (LI-COR, Lincoln, NE, USA, cat#927-50100) was used to block the membranes for 1 h at room temperature. An overnight incubation at 4 °C with primary antibodies was followed by a 1-h incubation at room temperature with secondary antibodies. The membranes were imaged on the Odyssey FC imager (LI-COR, Lincoln, NE, USA). Quantification of proteins was performed using Image Studio Lite software from LI-COR. Primary antibodies: p53 (Santa Cruz Biotechnologies sc-126); BST2 (Santa Cruz Biotechnology, Dallas, TX, USA, cat# sc-390719); hTERT (Abcam, Cambridge, UK, cat# ab183105); UCHL1 (Santa Cruz Biotechnology, Dallas, TX, USA, cat#sc-58593); Vinculin (Abcam, Cambridge, UK, cat# ab73412); GAPDH (Invitrogen, Waltham, MA, USA, cat# MA5-15738). Secondary antibodies included IRDye^®^ 680LT Goat anti-Mouse IgG (LI-COR, Lincoln, NE, USA, cat# 926-68020) and IRDye 800LT Goat anti-Rabbit IgG (LI-COR, Lincoln, NE, USA, cat# 926-32211)

## 3. Results

### 3.1. Lentivirus Transduction

Antibiotic selection verified the successful transduction of keratinocytes. All of the non-transduced control cells were dead within five days of exposure to antibiotics. Subsequently, RT-PCR confirmed the presence of E6 and E7 mRNA in each of the four biological replicates transduced with HPV16 E6 and E7 lentiviruses. Neither E6 nor E7 mRNA were detected in the LV-HEKn biological replicates.

**Figure 1:**
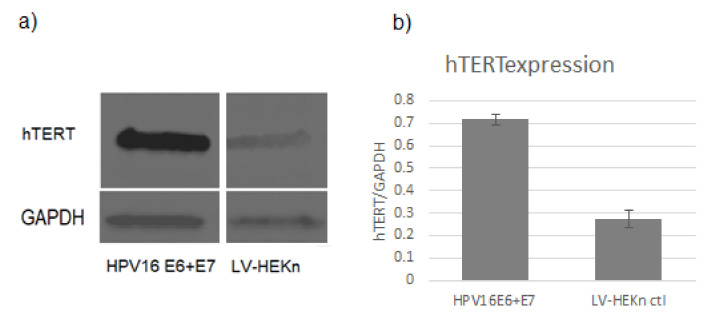
(**a**) Western blot experiments showed that hTERT levels were elevated in cells transduced with HPV16 E6 and E7 lentiviruses, compared to control cells (LV-HEKn). (**b**) Bar charts indicate the average level of hTERT normalized to GAPDH for three replicates of each cell line.

hTERT expression levels were assessed by Western Blot as a measure of immortalization and transformation capacity (Figure 1). Cells expressing both E6 and E7 were maintained in culture and continued to replicate beyond 100 population doublings; actively dividing for more than twice as long as the control cells. The cells expressing HPV16 E6 and E7 were not fully transformed as they did not show evidence of anchorage-independent growth, as determined by plating in soft agar, as previously described [27,28].

### 3.2. Identification of Significantly Differentially Expressed Proteins

The results of the LC-MS/MS analysis revealed 2454 out of 6906 proteins were significantly differentially expressed (adjusted *p*-value ≤ 0.02) between the cells expressing HPV16 E6 + E7 and the LV-HEKn control cells (Supplementary Table S1). Only the proteins significantly up or downregulated by more than 1.5-fold change were considered in the pathway analyses. There were 381 proteins upregulated by 1.5-fold (0.6 log_2_) change and 270 proteins downregulated by ≥−1.5-fold (−0.6 log_2_) change. The top 10 upregulated and the top 10 downregulated proteins are listed in Table 1 and Table 2 respectively.

At 19.6-fold change, LAMP3 was the protein with the most upregulated protein in E6 and E7 expressing cells compared to control (*p* = 0.0117). LAMP3, also known as DC-LAMP, is an integral membrane lysosomal-associated membrane protein whose function is not well defined. LAMP3 may be involved in cell survival during proteasomal inhibition [29,30]. Several proteins related to interferon signaling and immune response were among the top 10 upregulated proteins including, bone marrow stromal antigen 2 (BST2), HLA class I histocompatibility antigen IB-44 alpha chain (IB44), Tumor necrosis factor ligand superfamily member 10 (TNF10) and Interferon-induced GTP-binding protein MX2 (MX2).

Other notable proteins found to be upregulated in the E6 + E7 transduced cells were those associated with DNA replication, including members of the minichromosome maintenance complex (MCM 2-7), Sirtuin-1 (SIRT1) and midkine (MK).

The most downregulated protein was proline rich protein 9 (PRR9) with a −12.8-fold change (*p* = 2.14 × 10^−03^). PRR9 is involved in cell organization and differentiation, as are several of the other most substantially downregulated proteins: elafin (ELAF; *p* = 1.38 × 10^−03^), repetin (RPTN; *p* = 7.44 × 10^−03^), keratin 6B (K2C6B; *p* = 7.78 × 10^−04^), small proline rich protein (SPRR3; *p* = 1.80 × 10^−05^), involucrin (INVO; *p* = 1.51 × 10^−04^) and filamin (FILA; *p* = 1.56 × 10^−05^). Notably, INVO and RPTN were also among the top 20 down regulated proteins in the study by Xu et al. who used TMT-labeled proteomics analysis to determine proteomic dysregulation from cervical squamous cell carcinoma tissue compared to normal cervical tissues [17].

**Figure 2:**
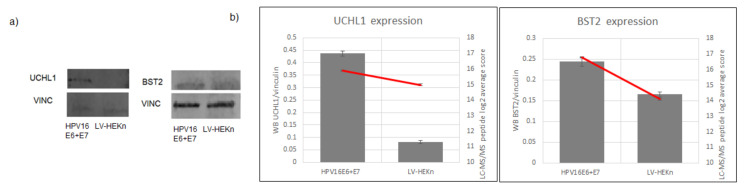
Protein levels in HPV16 E6 + E7 cells and LV-HEKn control cells displayed similar trends in Western blots and LC-MS/MS experiments. (**a**) Western blots of UCHL1 and BST2 in HPV16 E6 + E7 cells and LV-HEKn control cells. (**b**) The grey bars represent the abundance of protein measured by Western blot relative to the abundance of load control protein. The red line represents the LC-MS/MS measurements in the two cell lines. Bar charts indicate the average of three replicates of each cell line.

To validate the LC-MS/MS results we measured the amounts of two upregulated proteins in HPV E6 and E7 expressing cells by immunoblotting: Ubiquitin carboxyl-terminal hydrolase isozyme (UCHL1; *p* = 5.73 × 10^−05^) and bone marrow stromal antigen 2 (BST2; *p* = 4.91 × 10^−07^) were successfully visualized by western blot (Figure 2).

**Figure 3:**
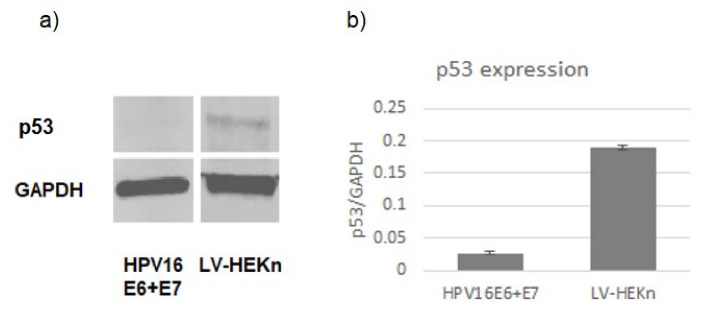
(**a**) Western blot experiments showed that p53 levels were reduced in cells transduced with HPV16 E6 and E7 lentiviruses compared to the control cells (LV-HEKn). (**b**) Bar charts indicate the average level of p53 normalized to GAPDH for three replicates of each cell line.

Although p53 was not detected by LC-MS/MS, Western blotting confirmed that p53 was downregulated in cells expressing HPV16 E6 and E7 (Figure 3). It is possible that p53 levels were below the limit of detection of LC-MS/MS because in unstressed cells levels of p53 are restricted by the Mdm2 E3 ubiquitin ligase, while in the presence of high-risk HPV E6 proteins p53 is degraded [31]. Alternatively, it is possible that the absence of p53 in the LC-MS/MS data was due to the limited number of trypsin cleavage sites in p53 that yield usable spectra, or the low abundance of multiple isoforms of p53 [32].

### 3.3. Network Analyses

IPA identified twenty-five networks and putative associated biological functions including Cell Cycle, DNA Replication, Recombination, and Repair, Cell Morphology, Cellular Movement, Cellular Assembly and Organization; Cell Death and Survival; Antimicrobial Response, Inflammatory Response, Immunological Disease; Cancer; and Dermatological Diseases and Conditions (Table 3).

**Figure 4:**
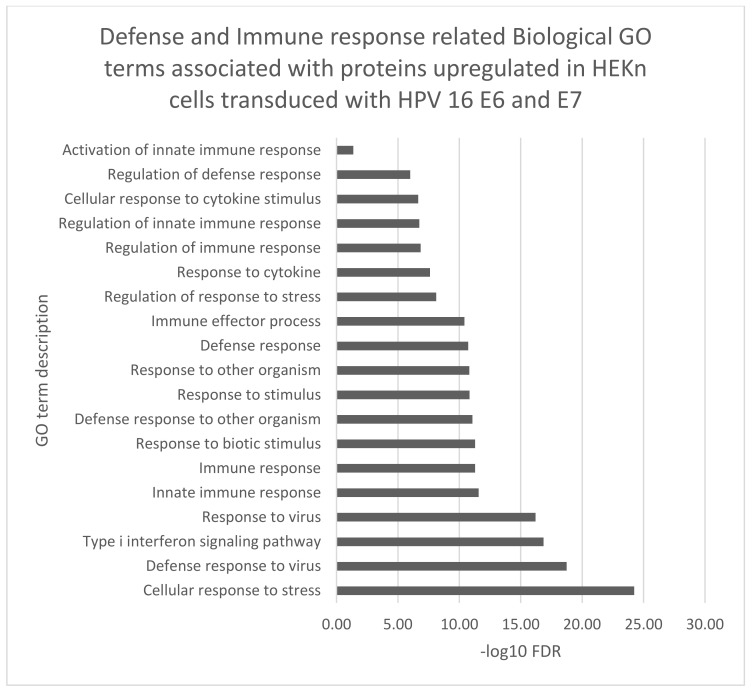
Biological GO terms related to defense immune responses were associated with the proteins with significantly increased expression (adjusted *p*-value ≤ 0.02) by ≥±1.5-fold change in keratinocytes transduced with HPV16 E6 and E7. FDR (False discovery rate).

**Figure 5:**
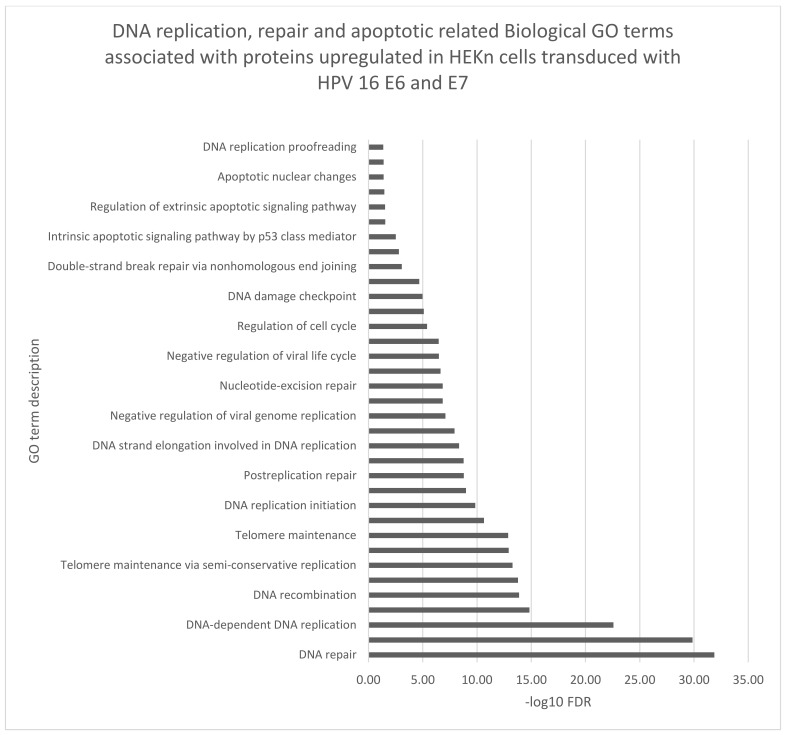
Biological GO terms related to DNA damage and apoptosis were associated with the proteins with significantly increased expression (adjusted *p*-value ≤ 0.02) by ≥±1.5-fold change in keratinocytes transduced with HPV16 E6 and E7. FDR (False discovery rate).

**Figure 6:**
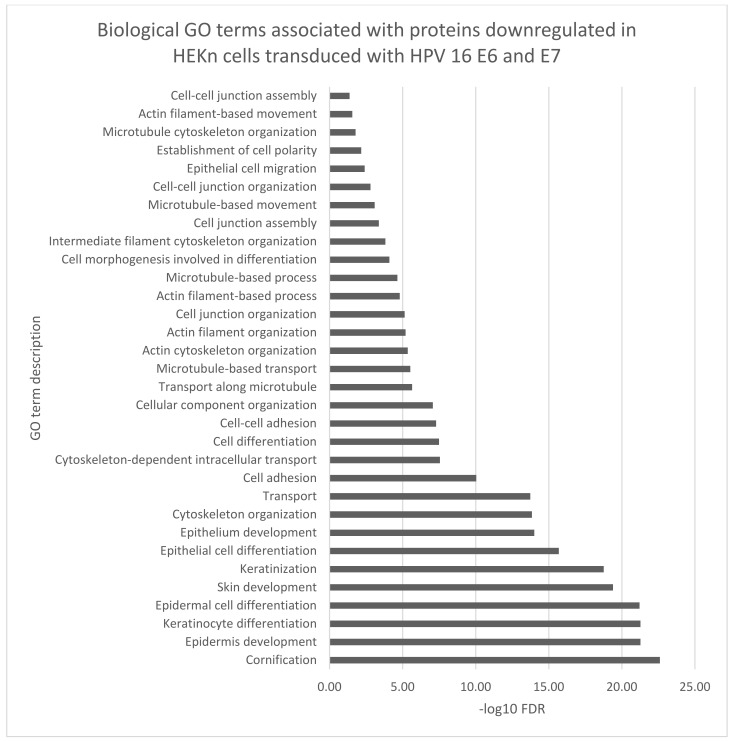
Thirty two- biological GO terms associated with epidermal differentiation, adhesion and cytoskeletal organization were predicted to be related to proteins with a reduced expression by ≥±1.5-fold change (adjusted *p*-value ≤ 0.02) in keratinocytes transduced with HPV16 E6 and E7 FDR (False discovery rate).

The biological function gene ontology (GO) terms assigned to our data using STRING software, corroborated many of the biological functions identified by IPA analyses (Figure 4, Figure 5 and Figure 6). Proteins which were either up or downregulated by ≥±1.5-fold change in keratinocytes transduced with HPV16 E6 and E7 were included in the analysis.

Several Biological GO terms associated with the proteins with significantly increased expression were related to immune response interferon response and inflammation. Proteins, such as 2′-5′-oligoadenylate synthase 1 (OAS1), signal transducer and activator of transcription 1 (STAT1) and E3 ISG15-protein ligase (HERC5) contributed to these GO term predictions.

DNA damage and apoptosis were predicted to be associated with proteins whose expression was significantly increased (adjusted *p*-value ≤ 0.02) by ≥±1.5-fold change in keratinocytes transduced with HPV16 E6 and E7 (Figure 4). Breast cancer type 1 susceptibility protein (BRCA1), DNA topoisomerase 2-alpha (TOP2A) and several histones (H1X, H10 H11, H12, H13) were among the proteins with significantly increased expression in our data. These proteins are predicted to influence apoptosis and DNA damage responses.

Finally, thirty-two biological GO terms associated with epidermal differentiation, keratinization and cytoskeletal organization were associated the proteins with significantly reduced expression (Figure 6). This group of significantly downregulated proteins includes several keratins, small proline rich proteins (SPR1A, SPR1B, SPRR2D, SPRR3, and SPRR4), desmocollins (DSC1, DSC2) which may influence the epidermal differentiation.

### 3.4. Canonical Pathway Analyses

**Figure 7:**
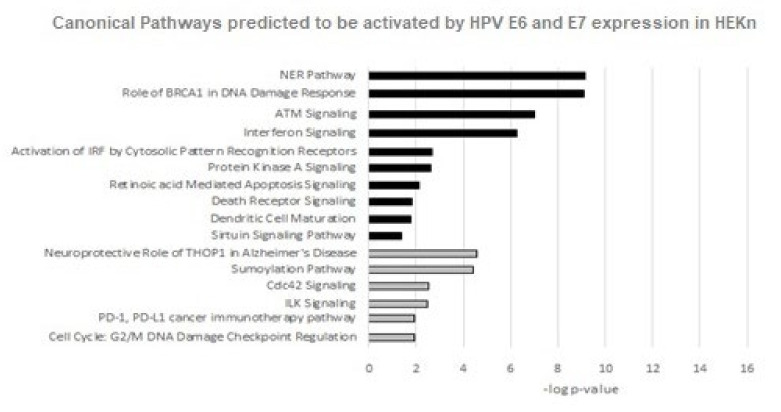
Canonical pathways predicted to be affected in HEKn cells expressing HPV16 E6 and E7 oncoproteins. Included in this analysis were proteins significantly differentially expressed (adjusted *p*-value ≤ 0.02) by ≥±1.5-fold change. Black shading indicates a predicted increase in activity and grey shading indicates a predicted inhibition of canonical pathway, based on current evidence in the Qiagen Ingenuity Pathway database.

IPA identified fifty-five canonical pathways predicted to be affected by the proteins significantly differentially expressed in HEKn cells transduced with HPV16 E6 and E7 (Figure 7). Understanding which pathways are affected by HPV oncoproteins may elucidate key host processes that are modulated by E6 and E7. We discovered that many of the altered canonical pathways were related to Cell cycle control, replication, Damage response, Antigen presentation pathway, Interferon signaling and apoptotic signaling.

For six of these pathways IPA predicted decreased activity: CDC42 signaling, Sumoylation Pathway, Cell Cycle: G2/M DNA Damage Checkpoint Regulation, Neuroprotective Role of THOP1 in Alzheimer’s Disease, PD-1,PD-L1 cancer immunotherapy pathway and ILK Signaling.

Similarly, there was enough evidence for IPA to predict the activation of ten canonical pathways: The Role of BRCA1 in Damage response, NER pathway, ATM signaling, Interferon signaling, Retinoic mediated apoptosis signaling, Activation of IRF by Cytosolic Pattern Recognition Receptors, Protein Kinase A Signaling, Death receptor signaling, Sirtuin Signaling pathway and Dendritic Cell Maturation.

The activation or inhibition of the other canonical pathways could not be predicted based on the current data in the IPA ingenuity database, but included pathways related to DNA damage, antiviral responses, cell survival and proliferation.

## 4. Discussion

### 4.1. Altered Protein Abundance in HEKn Expressing HPV16 E6 and E7

Persistent infection with high-risk HPV types is strongly associated with cancer. Although the mechanisms leading to transformation are not entirely elucidated, the persistent expression of E6 and E7 proteins of high-risk HPV types are known to actuate cell transformation [5,33,34,35,36]. Increased understanding of the proteins and pathways altered by the E6 and E7 oncoproteins should elucidate the mechanisms of viral persistence and the immune evasion tactics employed by HPV16 E6 and E7 preceding transformation. Of particular interest are the proteins or pathways that could be of therapeutic or prognostic value.

To determine the effects of the E6 and E7 oncoproteins on the host proteome, we performed LC shotgun proteomics analysis using HEKn transduced with lentiviruses containing HPV16 E6 and E7. One of this limitations of our study is that we did not verify the expression of the E6 and E7 by Western blot, however we did confirmed the presence of E6 and E7 mRNA by RT-PCR. Notably the cells expressing E6 and E7 were not transformed as demonstrated by soft agar assays, but did show altered expression of p53 and hTert. Interestingly, p16/CDKN2A, a cytological marker of transformation in HPV-induced cervical high-grade dysplasia, was not regulated in the presence of E6 and E7.

We elected to use a 2D HEKn culture model for this project because, despite its simplicity, it is appropriate for the investigation into effects of the viral proteins on the keratinocyte proteome. In vitro organotypic 3-D culture models are better suited for investigations into the HPV life cycle [37].

Our proteomic analysis identified 2454 significantly differentially expressed proteins (adjusted *p*-value ≤ 0.02). The subsequent pathway analyses were restricted to proteins significantly differentially expressed by ≥+1.5-fold change or ≤−1.5-fold change, which yielded a data set of 651 proteins which were subjected to pathway and network analyses.

There were a few recurring themes observed in our data, many of the upregulated proteins and their associated pathways were related to innate immune responses, interferon signaling and inflammation, as well as DNA replication, DNA damage response, cell proliferation, and cell death and survival. Among the downregulated proteins were those involved in epidermal differentiation.

### 4.2. HPV16 E6 and E7 Affect DNA Replication and DNA Damage Response Pathways

HPV must appropriate the host DNA replication machinery to amplify their viral genome. Although the HPV proteins E1 and E2 form the actual viral replication complex, the E6 and E7 oncoproteins have an essential, albeit indirect, role in viral replication. The binding of the tumor suppressor pRb by E7 allows the E2F transcription factor to activate DNA replication and there is increasing evidence that HPV also manipulates the DNA damage response pathways as part of its replication strategy [38]. The effects of HPV16 E6 and E7 on DNA replication and DNA damage responses are supported by our data, as several proteins and canonical pathways related to activation of DNA replication and DNA damage responses were found to be deregulated in HEKn expressing HPV16 E6 and E7 proteins.

Mini-Chromosome Maintenance proteins (MCM), make up an essential component of the DNA pre-replication complex. As part of that complex, six MCM proteins (MCM2–MCM7) form a hexamer which together with cell division cycle 6 (CDC6) and Chromatin Licensing And DNA Replication Factor 1 (CDT1) binds to a DNA origin of replication and initiates DNA replication [39]. Typically, levels of MCM proteins are only increased during the S-phase of the cell cycle. MCM proteins are strongly associated with cell proliferation and their deregulation has been recognized as significant prognostic biomarkers for several types of cancer, including cervical cancer [40,41,42]. In our data, MCM2-7 were all upregulated in the HEKn cells expressing HPV16 E6 and E7 in agreement with a previous study which documented the upregulation of MCM2-7 mRNA in cervical cancer and precancerous lesions [41]. The mechanisms responsible for the elevated levels of MCM in cervical cancers are not well described, but in lung cancer, YAP/TAZ expression has been cited for promoting the upregulation of MCM7 transcription leading to cell proliferation [43]. YAP and TAZ proteins are key regulators of HIPPO signaling pathway, but neither of these proteins, nor the HIPPO signaling pathway, was found to be significantly differentially altered in IPA analyses between HPV16 E6 and E7 expressing HEKn cells and control cells. It is likely that in the cells expressing HPV16 E6 and E7, the increase in MCM proteins is related to the repression of pRb by E7 because pRb loss or repression is known to permit cyclin E1, a target of pRb, to recruit MCM proteins to pre-replication complexes [44].

Ataxia-telangiectasia mutated (ATM) signaling and DNA Double-Strand Break Repair by Homologous Recombination were among the canonical pathways predicted to be significantly affected in the cells expressing HPV E6 and E7. Typically, when the DNA of a cell is damaged, one of the cellular DNA damage response pathways, ATM or ataxia-telangiectasia mutated Rad3-Related (ATR), becomes activated arresting the cell cycle until the damaged is repaired. Intriguingly, it appears that HPV replication promotes the activation of DNA damage repair signaling and HPV is not unique in this approach to viral DNA replication. The polyomavirus simian virus 40 (SV-40) and several herpesviruses also activate the ATM and recruit DNA repair factors for viral replication [45,46,47]. Interestingly, using LC-MS/MS, Guzel et al. also recently reported that DNA repair pathways were altered in early stages of cervical cancer [14].

Two members of the ATM pathway significantly upregulated in our data associated with HPV16 E6 and E7 expression include Breast cancer type 1 susceptibility protein (BRCA1) (+2.8-fold *p* = 0.0071) and the histone H2AX (+1.8-fold *p* = 0.024).

Upon detection of DNA damage, the histone H2Ax locates to the site of damage and is phosphorylated by ATM to ɣ-H2AX. Together with other members of the DNA damage response, BRCA1 and ɣ-H2AX activate cell cycles checkpoints to arrest the cell cycle and initiate homologous recombination or non-homologous end-joining DNA repair. Being a tumor suppressor, it is interesting that HPV E6 and E7 would promote BRCA1 expression, but remarkably, these viral oncoproteins have evolved a mechanism to bind and functionally inactivate BRCA1 although they do not proteolytically degrade it [48]. Via their zinc finger domains, E6 and E7 prevent BRCA1 from repressing the estrogen-receptor-α (ER-α) transcriptional activity and prevent the inhibition of cMyc transactivation by BRCA1 [48].

### 4.3. Immune Evasion and Interferon Signaling Aberration by HPV16 E6 and E7

Modification of protein acetylation is one mechanism by which HPV is known to evade the host’s immune system and promote viral DNA replication [49,50,51]. Here we have proteomic evidence documenting the differential levels of protein deacetylases related to HPV16 E6 and E7 expression. Among the upregulated proteins from HEKn cells expressing HPV16 E6 and E7, there were three protein deacetylases, HDAC2 and HDAC7, and sirtuin (SIRT1) which were upregulated by 1.4, 1.5, and 1.7-fold change respectively. Histone deacetylases (HDAC) and SIRT1 deacetylases, also known as NAD-dependent deacetylase sirtuin-1 have previously been shown to be deregulated in cancers, including HPV associated cancers [49,50,52,53,54]. Interestingly, elevated levels of HDAC and SIRT1 have been linked to HPV immune evasion strategies. For example, HPV E7 reduces TLR9 expression by recruiting HDAC2 to the TLR9 regulatory region, ultimately repressing interferon responses [51]. Although there is recent evidence that SIRT1 represses the AIM2 (absent in melanoma inflammasome) innate immune response to infection by DNA viruses, promoting survival and proliferation of HPV positive cells, there is no evidence of the downregulation of AIM2 associated proteins such as interleukin-1β or caspase among our list of significantly differentiated proteins [54]. SIRT1 can target p53 for deacetylation, which would presumably enhance the abrogation of the p53 pro-apoptotic signaling pathway promoted by HPV E6.

In addition to immune evasion, SIRT1 is required for HPV viral replication. SIRT1 forms a complex with HPV E1 and E2 proteins at the HPV origin of replication, deacetylates E2 and recruits the DNA repair protein Werner helicase [38,49,55]. Fittingly, Werner helicase interacting protein 1 was observed in our data to be upregulated in HEKn cells expressing E6 and E7.

The fidelity of HPV replication also relies on SIRT1. Knockdown of SIRT1 in HPV16 infected cells results in a highly mutated genome due to the failure to recruit to the E1/E2 complex [49,55]. Furthermore, SIRT1 knockdown in HPV31 infected cells diminishes the number of viral genomes in the host cells [50].

Interferon signaling and antiviral responses were among the biological functions and canonical pathways, which IPA predicted to be altered in HEKn cells expressing HPV16 E6 and E7. Production of type 1 Interferon results from the detection of viral DNA by host pattern recognition receptors and the initiation of the innate immune signaling. One of the proteins that likely contributed to the activation of interferon signaling and antiviral responses predicted for our data is LAMP3. Previous studies have associated LAMP3 with increased levels of interferon-dependent anti-viral genes [56].

Our findings agree with a previous study, where LAMP3 mRNA was found to be highly expressed in 100% of cervical cancers [57]. LAMP3 overexpression is highly associated with metastatic potential and it has been proposed that LAMP3 may be an important prognostic biomarker for cervical cancer [57]. In addition, high levels of LAMP3 have been identified as prognostic biomarkers in esophageal squamous cell carcinoma, hepatic and ovarian cancers [58,59,60].

LAMP3, also known as DC-LAMP, is an integral membrane lysosomal-associated membrane protein whose role is not well defined. There is some evidence that LAMP3 is required for cell survival during proteasomal inhibition and there have been some recent investigations into the role of LAMP3 in different cancers [29,30]. For example, in *osteosarcoma cells*, *LAMP3* significantly increased cell viability and abrogated apoptosis by regulating *p53* expression [30]. The role of LAMP3 has also been explored in HPV positive cells. In HeLa cells, LAMP3 is associated with increased levels of interferon-dependent anti-viral genes: STAT-1, IRF7, HERC5, ISG20 and OAS1; silencing of LAMP3 results in their downregulation [56]. Fittingly, in our data STAT-1, HERC-5 and OAS1 were upregulated in the cells expressing HPV16 E6 and E7 proteins.

Another significantly upregulated protein linked to interferon expression in cells expressing HPV16 E6 and E7 is Bone Marrow Stromal Cell Antigen (BST2). While BST2 is induced by interferon, it also negatively regulates interferon by binding with the Immunoglobulin-like transcript 7 in plasmacytoid dendritic cells [61,62]. The ability to modulate interferon levels would presumably limit the negative consequences of protracted interferon exposure on the host.

An increase in BST2 has been reported in several types of cancer including cervical cancer [63,64,65,66,67,68]. The overexpression of BST2 in breast cancer cells is associated with enhanced cell proliferation and inhibition of apoptosis [69]. BST2 enhances survival through proteasome-mediated degradation of the proapoptotic protein BIM (Bcl-2 like protein 11), a member of the Bcl-2 family. Mahauad-Fernandez and Okeoma recently described a mechanism by which BST2 dimerization activates ERK1/2 and phosphorylates and degrades BIM, preventing anoikis in breast cancer cells [70]. BIM was not significantly differentially expressed in our data however.

HPV16 E6 can bind the interferon regulatory factors, IRF3 and IRF9, to thwart interferon signaling [71]. Although we did not find IRF3 or IRF9 to be differentially expressed, we did observe that UCHL1 (Ubiquitin carboxy-terminal hydrolase L1), a protein known to affect IRF3 phosphorylation, was upregulated by 1.9-fold change in HEKn cells expressing HPV16 E6 and E7 [72]. UCHL1 is a deubiquitinating enzyme that prevents the polyubiquitination of tumor necrosis factor 3 (TRAF3) and also results in the degradation of NEMO, a modulator of NFkB (nuclear factor kappa-light-chain-enhancer of activated B cells) signaling [72]. NFkB signaling regulates cytokine production and cell survival signaling, and its dysregulation has long been correlated to cancers [73].

Midkine, a heparin-binding cytokine that promotes cell proliferation and migration, was among the top 10 most upregulated proteins in HEKn cells expressing HPV16 E6 and E7. Midkine has previously been reported to be upregulated in cervical, bladder, esophageal and gastric cancers and increased levels of midkine are associated with poor prognosis [74,75,76,77]. A previous study explored midkine’s effects on growth and survival. That group added midkine to the medium of cultured neuronal cells and found that this resulted in activated MAPK and PI3K signaling, while caspase-3 was inhibited [78]. It was subsequently discovered that midkine promotes cell proliferation and survival by phosphorylating the ALK (anaplastic lymphoma receptor kinase) in fibroblasts leading to MAPK and PI3K activation [79]. Activation of MAPK and PI3K does not fit with our data however as MAPK is predicted to be strongly inhibited in our cells expressing HPV16 E6 and E7. The implications of midkine upregulation in HEKn cells expressing HPV16 E6 and E7 remains to be explored and may be worth pursuing, as midkine has garnered attention as a therapeutic target for one type of lung cancer [80].

### 4.4. Dysregulation of the Keratinized Barrier by HPV16 E6 and E7 Expression

The third biological trend highlighted in our proteomic analyses was dysregulation of epidermal differentiation and the inhibition of the formation of the keratinized barrier. These biological functions were predicted to be significantly downregulated in HEKn cells expressing HPV16 E6 and E7. Seven of the top ten downregulated proteins in the HPV16 E6 and E7 cell line were associated with epidermal differentiation: Proline-rich protein 9, elafin, repetin, keratin type II, small proline-rich protein 3, involucrin and filaggrin. Interestingly, a previous gene expression study also found several genes associated with keratinocyte differentiation to be downregulated in keratinocytes expressing high-risk E6 proteins [81]. In that study, genes encoding elafin, small proline-rich proteins, keratins and involucrin were among the differentiation-associated genes significantly affected [81].

The keratinized layer of the epithelium acts as a mechanical and permeability barrier, and so its inhibition by HPV might be essential to foster the egress of viral particles from the host. Co-incidentally, the inhibition of differentiation is a hallmark of cell transformation. Normally, primary keratinocytes undergo an irreversible differentiation process forming a keratinized envelope when deprived of anchorage to a semi-solid medium [82]. Malignant cells, on the other hand, fail to differentiate and do not form a keratinized envelope when cultured in an anchorage-independent manner [82]. Despite the downregulation of the proteins associated with epidermal differentiation in our cells, when grown on soft agar there was no evidence of colony formation.

## 5. Conclusions

Using LCTMT-labelling and shotgun proteomics with pathway analysis we have catalogued proteomic signatures significantly differentially expressed in HEKn cells expressing HPV16 E6 and E7 proteins. Our data support previous gene expression studies linking HPV to the manipulation of the host’s DNA damage response pathways, DNA replication and interferon signaling. We have shown that several DNA damage response proteins and interferon modulating proteins including BRCA1, H2AX, SIRT1 and LAMP3 are unambiguously upregulated in primary keratinocytes expressing HPV16 E6 and E7 oncoproteins. Our study also identified proteins, such as the heparin-binding protein midkine and the ubiquitinase UCHL1, to be upregulated. These proteins warrant further exploration regarding their relationship with HPV infection.

Notably, some of these significantly differentially up-regulated proteins have potential as biomarkers or for the development of therapies. For example, LAMP3, which was the most upregulated protein in the HEKn cells transduced with HPV16 E6 and E7, is regarded as a biomarker of several cancers [58,59,60]. Further investigation into LAMP3 expression in various stages of cancers is needed to determine the prognostic value of this protein. Midkine is also interesting as a possible therapeutic target; not only is midkine considered a prognostic indicator for various cancers, but it has also garnered attention as a therapeutic target. An inhibitor of midkine has recently been described as a potential mesothelioma treatment [80].

Future studies could include validating the clinical implications of these findings in vivo using CIN lesions or determining if any of these differentially expressed proteins or deregulated pathways may be useful as therapeutic targets in HPV related cancers or for the identification of biomarkers for HPV prognosis. Although there are vaccines targeting several of the high-risk types of HPV, there are millions of people persistently infected with HPV. For these people, non-surgical therapeutic options are lacking. Further characterization of the proteins or pathways identified in this study will advance our understanding of the mechanisms of HPV16 E6 and E7 mediated immortalization and transformation of keratinocytes.

## Figures and Tables

**Table 1:** List of the top 10 significant proteins most increased in abundance as measured by mass spectrometry-based shotgun proteomics analysis of HEKn cells expressing HPV16 E6 and E7 compared to control cells.

Protein Name	UNIPROT_ID	Fold Change	Adjusted *p*-Value
Lysosome-associated membrane glycoprotein 3	LAMP3_HUMAN	19.6	1.17 × 10^2^
Histone H1.1	H11_HUMAN	8.5	6.80 × 10^4^
Bone marrow stromal antigen 2	BST2_HUMAN	6.4	4.91 × 10^7^
HLA class I histocompatibility antigen, B-44 alpha chain	1B44_HUMAN	5.6	3.38 × 10^7^
Solute carrier family 43 member 3	S43A3_HUMAN	5.0	3.13 × 10^4^
Midkine	MK_HUMAN	4.3	1.65 × 10^3^
Neurofilament heavy polypeptide	NFH_HUMAN	4.3	2.79 × 10^4^
Histone H1.3	H13_HUMAN	4.1	1.76 × 10^2^
Interferon-induced GTP-binding protein Mx2	MX2_HUMAN	4.1	2.68 × 10^7^
Tumor necrosis factor ligand superfamily member 10	TNF10_HUMAN	4.0	3.46 × 10^6^

**Table 2:** List of the top 10 significant proteins most decreased in abundance as measured by mass spectrometry-based shotgun proteomics analysis of HEKn cells expressing HPV16 E6 and E7 compared to control cells.

Protein Name	UNIPROT_ID	Fold Change	Adjusted *p*-Value
Proline-rich protein 9	PRR9_HUMAN	−12.8	2.14 × 10^3^
Elafin	ELAF_HUMAN	−8.1	1.38 × 10^3^
Repetin	RPTN_HUMAN	−6.8	7.44 × 10^3^
Keratin, type II cytoskeletal 6B	K2C6B_HUMAN	−6.0	7.78 × 10^4^
Insulin growth factor-like family member 1	IGFL1_HUMAN	−5.2	1.10 × 10^3^
Small proline-rich protein 3	SPRR3_HUMAN	−4.5	1.80 × 10^5^
Aldo-keto reductase family 1 member C3	AK1C3_HUMAN	−4.4	3.82 × 10^4^
Involucrin	INVO_HUMAN	−4.2	1.51 × 10^4^
Filaggrin	FILA_HUMAN	−4.1	1.56 × 10^5^
Alpha-2-macroglobulin-like protein 1	A2ML1_HUMAN	−4.0	5.40 × 10^4^

**Table 3:** Twenty-five protein networks were identified by IPA based on the proteins differentially altered in abundance by ≥±1.5-fold change in cells expressing HPV16 E6 and E7 compared to control cells. In addition to up and down regulated molecules, proteins predicted by IPA to participate in a particular network, although not detected by our LC-MS/MS experiments, are also listed. The score reflects the probability of finding the observed proteins in a given network by random chance. A higher score value reflects a lower probability. The top diseases and functions associated with proteins in each network are listed. Multiple networks may have similar biological functions or disease predictions.

Score	Molecules in Network			Top Diseases and Functions
	Upregulated	Downregulated	Not Detected	
49	BTN3A2, BTN3A3, CFAP20, EZH2, FBLN1, HLTF, KIF14, LAMP3, MCM2, MKI67, MUC1, NOLC1, NT5E, PLEKHA4, PSPC1, RBMX, RIF1, UBR7, WBP4	AMACR, ARL8B, CRCT1, EFNB2, FLG, FTH1, KIF5B, MFAP5, POF1B, RBM3, SLC16A2, SNX18	Alp, Focal adhesion kinase, GTPase, Secretase gamma	Cancer, Organismal Injury and Abnormalities, Reproductive System Disease
44	BUD13, CMSS1, EPSTI1, HAT1, NASP, NOP14, PRKRIP1, RBMX2, SLC43A3, TOP1, TRIM22, USP11, VEZF1	ACAA1, ATP6V0A1, ATP6V1B2, ATP6V1G1, CAMSAP3, CNN2, FAM160A1, HOOK3, KIAA0930, OSBPL9, PDLIM1, SERPINB7, SLC2A6, TRAPPC9, VPS41, VPS8	AP-3, NFkB (complex), Topoisomerase, Vacuolar H+ ATPase, peptidase, tryptase	Cellular Compromise, Cellular Movement, Hematological System Development and Function
44	BRIX1, DDX21, DDX27, DDX60L, EBNA1BP2, FTSJ3, H1F0, HIST1H1A, HIST1H1C, HIST1H1E, HMGN2, HNRNPU, IFI16, LYAR, PPM1G, PSIP1, PTMA, RBM28, REXO4, RNF169, RNF213, RRS1, RSL1D1, SURF6, UBTF	HMGCR, MAP2, TNKS1BP1	PP1 protein complex group, PPAN, phosphatase, Histone H1, Gsk3, P-TEFb, Pp2c	Developmental Disorder, Hereditary Disorder, Infectious Diseases
44	ARGLU1, CCDC137, CFDP1, DDX49, EXOSC2, EXOSC3, EXOSC5, EXOSC6, EXOSC8, EXOSC9, GLB1L, LAP3, MASTL, MPHOSPH6, SNRNP70, SP140L, SPOUT1, UTP23, ZCCHC8, ZNF740	APP, C1orf116, CLIC3, CLN5, EOGT, LAD1, RETSAT, SCEL, SH3BGRL3, SPRR4, TOM1L2	Pi3k class III, Rnr, plasminogen activator, succinate dehydrogenase	RNA Post-Transcriptional Modification, Developmental Disorder, Hereditary Disorder
41	FBXO6, GINS3, GMPS, GUSB, HIST1H1D, LY75, MCM3, MCM4, MCM5, MCM6, MCM7, NOC3L, NRCAM, ORC3, ORC4, ORC5, SSRP1, TONSL, UHRF1, WARS	DCBLD2, IGSF3, MAN2B1, MAN2B2, MANBA, RNASET2, SGSH, SIAE	E2f, Mannosidase Alpha, Mcm, ORC, RPA, TIP60, Vegf	DNA Replication, Recombination, and Repair, Cell Cycle, Carbohydrate Metabolism
40	KIF23, KRT19, MCAM, MTHFD2	DSC1, DSC2, DSG1, DSG3, DSP, EVPL, FLG2, FTL, JUP, KLC3, KRT10, KRT13, KRT15, KRT16, KRT17, KRT23, KRT3, KRT6A, KRT6B, KRT6C, KRT78, KRT80, PKP1	Alpha catenin, Cytokeratin, Irp, KRT5/6, Keratin, Keratin II, 6,PI3K(complex), PRKAA	Cell Morphology, Embryonic Development, Hair and Skin Development and Function
37	ANLN, CIT, NUB1, RACGAP1, TLK1	ARPC3, CALD1, CALML3, CAP1, CORO1A, CORO1B, EEF1B2, ENAH, FAT1, HOMER3, MYH9, MYL12A, MYL6, MYO5A, PLBD2, PLCD1, SLC9A3R1, TPM1, TPM3, TPM4, VAT1	Actin, Cofilin, Erm, F Actin, G-Actin, Lamin b, Myosin, Pkc(s), Tropomyosin	Cellular Assembly and Organization, Cellular Function and Maintenance, Cellular Movement
36	ADAR, CBX4, CMPK2, DDX60, IFIT3, MX1, MX2, N4BP1, OASL, PLSCR1, PML, PNPT1,RSAD2, SAMD9L, SMC5, SMC6, SMCHD1, SP110, STAT1, SUMO1, SUMO3, TGM2	CA12, DHRS1, RPLP1, SPRR1A	IFN Beta, IFN alpha/beta, IL-2R, IL23, Ifn, JAK, JAK1/2, SUMO, cytokine	Antimicrobial Response, Inflammatory Response, Infectious Diseases
31	APOL2, BRCA2, CYP1B1, DCD, DDX50, ESRRA, GRPEL2, HELZ2, HNRNPC, HNRNPH3, IGFBP4, LGALS3BP, LSM8, NCAPG2, NCAPH2, PRPF19, SMC4, TFAM, TOP2A, TRIM14	NDUFA4L2, PDLIM5, UBE2E3	BRCA1-BRCA2-FANCD2-FANCN-RAD51, Collagen type V, Cytochrome bc1, Fanc, LRP, Mitochondrial complex 1, P glycoprotein, PROTEASE, SRC (family)UBE2, cytochrome-c oxidase, snRNP	Cell Cycle, Cellular Assembly and Organization, DNA Replication, Recombination, and Repair
29	ARID5B, COL1A2, COL6A1, COL6A2, CTSK, MBD3, MMP9, P3H3, P3H4, PDS5B, RCN3, ZNF768	CAST, CCN1, ITGAV, SDC1, SERPINB13, SERPINB5, SERPINE2, SFRP1, TMBIM1, WNK1	Cathepsin, Fgf, Fibrinogen, Growth hormone, Integrin, Mi2, Mmp, RNA polymerase I, Smad, TFIIH, Tgf beta, calpain, collagen	Tissue Development, Organismal Injury and Abnormalities, Connective Tissue Development and Function
29	GBP1, HMGN3, MSLN, MYEF2, NBR1, PLAT, SLC29A1, SRSF9, UNC93B1, ZC3H4	CPA4, CROT, DPP4, FAP, HTRA1, KLK7, RPS27L, S100A10, SLC20A1, SLPI, SPINK5, TNC, TP53I3	Collagen type I, Collagen type II, Cpla2, Fc gamma receptor, Fibrin, Kallikrein, P38 MAPK, SYK/ZAP, Serine Protease, Thrombospondin, growth factor, trypsin	Tissue Development, Cell Death and Survival, Cardiovascular Disease
28	KIF2C, KIF4A, NEFH, NUMA1, SMC1A, SMC2, SMC3, STMN1, UBE2L6	ACTR1A, AHNAK2, ALDH1A3, CLIP1, DCTN1, DCTN2, DCTN3, DYNC1I2, GM2A, MAPRE3, ROBO1, TUBB2A	Alpha tubulin, BETA TUBULIN, Dynein, Gamma tubulin, MIR101, Mapk, NCK, PDGF-AA, Pak, Pdgf Ab, SMC, Sos, tubulin (family)	Cellular Assembly and Organization, Cellular Function and Maintenance, Tissue Development
28	BAG2, DNAJC9, HERC5, HNRNPD, HSF1, HSPA2, NSMCE4A, SLC1A5, SPIN1	BAG3, CRYAB, CSTA, DNAJC5, FILIP1L, HSPB1, MAP1B, PI3, PSTPIP2, SBSN, SEMA3C, TRIAP1, TUBA4A	26s Proteasome, Calcineurin protein(s), Calmodulin, Complement, HSP, Hdac, Hsp70, Hsp90, MHC Class II (complex), PP2A, Syntaxin, TCF, caspase	Post-Translational Modification, Protein Folding, Cellular Compromise
27	ANP32B, DHTKD1, EPCAM, FUT8, ICAM1, ISG15, MGME1, RAVER1, RCOR3, TREX1, VRK1	ACTN1, ACTN4, ASAH1, FABP5, LGALS7/LGALS7B, MVP, PALLD, PDLIM4, PLD1, SLC38A7	ATPase, Alpha Actinin, Ap2, Casein, Ferritin, GOT, IgG, IgG2a, IgG2b, Igg3, MHC, SCAVENGER receptor CLASS A, hemoglobin, transglutaminase	Cellular Assembly and Organization, Glomerular Injury, Inflammatory Disease
27	ATF1, CHTOP, DCK, ERH, SAMHD1, SRSF4, TRPV2, ZMYND11	AKAP13, ALS2, APPL1, CALML5, CAMK2G, CDA, DYSF, KIF1C, MAP4, PRKAR2A, RAB5B, SFN, SMPDL3A	14-3-3, Camk, Cdc2, Cyclin B, Insulin, MAP2K1/2, MTORC2, NFkB (family), Nos, Pde, Pka catalytic subunit, Rab5, Raf, SL1	DNA Replication, Recombination, and Repair, Nucleic Acid Metabolism, Small Molecule Biochemistry
27	ACOT4, BRCA1, CDK1, DNMT1, FKBP10, H2AFX, HMGA1, HNRNPR, LMNB1, OGFR, PARP1, PDIA4, PHGDH, TCEA2, TMPO, TTF1	CSDE1, EPN3, PAM, SDK2, SPRR2D	CD3, Ck2, G protein beta gamma, Histone h3, Holo RNA polymerase II, ITPR, NMDA Receptor, PLC gamma, Pdgfr, Ppp2c, STAT, Sfk, TCR, tubulin	Cellular Assembly and Organization, Cell Death and Survival, Embryonic Development
26	CGAS, LIG1, NDFIP1, OAS1, OAS2, OAS3, POLD1, POLD2, POLD3, POLE, POLE3, PRIM1, PRIM2, RFC2, WDHD1	CDH13, GPNMB, MFGE8, PIK3IP1, SCD5, SPRR3	Akt, Cadherin, Collagen Alpha1,Collagen type VI, DNA Polymerase, DNA polymerase delta, DNA polymerase epsilon, DNA-PK, DNA-directed DNA polymerase, Fascin, Oas, POLbeta-POLepsilon-POLgamma-XRCC1-LIGI-PARP1-PCNA-FEN1, POLbeta-POLgamma-POLepsilon-XRCC1-LIGI-PARP1, nucleotidyltransferase	Cancer, Hematological Disease, Immunological Disease
26	CD3EAP, CHTF18, DSCC1, FANCI, FEN1, FHL1, IRF2BPL, MDC1, NBN, PCNA, PPHLN1, RBM39, RPA1, RPA2, SUPT16H, TCOF1, TXNIP, UHRF2	AHNAK, ANK3, DOCK9, KIF13A, LACTB	CaMKII, Cdk, Cyclin A, Fcer1, NADPH oxidase, NFAT (complex), Nfat (family), PI3K (family), PI3K p85, Rac, Rb, TSH	DNA Replication, Recombination, and Repair, Cellular Assembly and Organization, Cellular Function and Maintenance
25	CSPG4, H1FX, HERC6, MBD4, NES, PMAIP1, TYMP, UCHL1, USP28	A2ML1, DMKN, FBLIM1, HAS3, ITGB6, LAMP2, MICALL2, SLC25A20, TRIM29, VPS29, VPS35	Collagen type III, Collagen type IV, Collagen(s), Complement component 1, DUB, ERK, Filamin, GPIIB-IIIA, Laminin (complex), Laminin1,RAB7, S100, chymotrypsin, elastase, gelatinase	Hereditary Disorder, Neurological Disease, Organismal Injury and Abnormalities
25	BAZ1B, CBX1, CBX3, CBX5, CHAF1A, CHAF1B, DSN1, HP1BP3, LBR, MIS12, MSH6, NT5C3A, PMF1/PMF1-BGLAP, RFC1, RFC4, RFC5, SP100	EXOC7,SLC1A3,SPRR1B	BRCA1 complex B, Basc, CAF-1Fgfr, H-2db, HP1, Hspg, L-type Calcium Channel, Ldh (complex), MRN, MutS alpha, PCNA-CAF1, Pde4, Pka, Rfc	Cellular Development, Cellular Growth and Proliferation, Embryonic Development
23	CFB, DDX58, DTX3L, EIF2AK2, IFI35, IFIT1, MACROD1, MLH1, NMI, PARP12, PARP14, PARP2, PARP9, RNF114, TDRD7, TNFAIP3, TNFSF10	NPC2, TPRG1L	ATM/ATR, H2AF, Hsp27, I kappa b kinase, IFI35, Ikb, Ikk (family), Inflammasome (Nalp3, Asc, Casp1), MLH1-MSH2-MSH6-PMS2, PARP, Ras homolog, Tlr, Tnf (family), Tnf receptor, cytochrome C, mismatch repair, pentosyltransferase	Connective Tissue Disorders, Immunological Disease, Inflammatory Disease
23	ASNS, CCPG1, DHRS13, DHX40, FANCD2, HLA-A, INA, RBCK1, RNPS1, SIRT1, SPAG7, SUZ12, TNIP1	DMKN, EEF1G, GAN, RPTN, S100A8, YWHAG	CNTN6, Csprs (includes others), EGLN, H2-Q8, MTORC1, MYCT1, NSMF, Nc2, PRSS27, RAB20, RNA polymerase II, SMTNL2, Sod, TNIP1, Ubiquitin, VWA3B, chemokine	Connective Tissue Disorders, Dermatological Diseases and Conditions, Immunological Disease
21	B2M, BST2, ERAP1, HLA-B, HLA-C, HLA-E, HLA-F, HLA-G, H, MAT2B, PSMB10, PSMB9, PSME1, PSME2, TAP1, TAP2, TAPBP, TAX1BP1	SERPINB3	20s proteasome, ERK1/2, HLA Class I, HLA-B27, Hla-abc, Ifnar, Immunoproteasome Pa28/20s, KIR, MAP1LC3, MHC (Major Histocompatibility Complex), MHC CLASS I (family), MHC Class I (complex), MHC I-α, PKC alpha/beta, Stat1-Stat2, Tap, peptide-Tap1-Tap2	Antigen Presentation, Protein Synthesis, Cellular Function and Maintenance
17	ANP32E, CIR1, FKBP10, LIN54, MELTF, SLC25A35, SMOC1, VPS72	CYB5R1, DSC2, LAD1, MEGF9, SERF2, ZNF185	C11orf52, DENND5B, Dynamin, ESR2, GABPB2, HIF1AN, HNF4A, L2HGDH, MMP17, NUPR1, OSER1, PHTF1, SMOC1, SPIRE1, THAP4, TMEM50B, TMEM53, TNF, TRIM25, TSNAXIP1, UTP11	Tissue Development, Cell Morphology, Cellular Compromise
17	ANP32B, FBLN1, HLA-B, MCAM, NRCAM	ANO10, CAP1, CLCA2, CNN2, GLTP, HTRA1, KCNK6, MPV17L2, SEMA3C, SNX30	AQP3, CD99,C EMIP2, EMG1, ESR1, FN1, HYAL1, KHDRBS1, MYLK2, NDC1, NUP88, OSTF1, RPS3, SLC26A2, SLC44A1, TCF/LEF, TCF7L2, TNS3, TWIST, UGT8	Cellular Movement, Hematological System Development and Function, Immune Cell Trafficking

## Data Availability

The data presented in this study are available on request from the corresponding author. The data are not publicly available due to institutional policies at the Public Health Agency of Canada.

## Supplementary Materials

The following supporting information can be downloaded at: https://www.mdpi.com/article/10.3390/v14081764/s1, Table S1: Significantly differentially expressed proteins (adjusted *p*-value ≤ 0.02) between the cells expressing HPV16 E6 + E7 and the LV-HEKn control cells.
